# Aspects of power sensitivity among social workers and social work students: a comparative study

**DOI:** 10.3389/fmed.2025.1580234

**Published:** 2025-08-25

**Authors:** Melanie Misamer, Claudia Bartels, Michael Belz

**Affiliations:** ^1^HAWK University of Applied Sciences and Arts, Göttingen, Germany; ^2^Department of Psychiatry and Psychotherapy, University Medical Center Göttingen, Göttingen, Germany

**Keywords:** power sensitivity, constructive use of power, destructive use of power, social work professionals, social work students, self-serving bias

## Abstract

The asymmetrical nature of the relationship between social workers and their clients may lead to abuse of power due to a human trait or corruption. A high level of power sensitivity is thus crucial to counteract power abuse. Ideally, this topic should be covered during studies, as the risk of corruption rises with everyday working life. In this study, we aimed to assess basic and specific aspects of power sensitivity both for 271 students and 414 professionals, covering (1) general differences for the total sample, (2) differences between both groups and (3) differences between subgroups (semesters, professional years, field of profession; ratings from 0 to 100%). While importance of power sensitivity (94.7%) and professional ethics/principles (91.9%) were rated higher than all other items (*p* < 0.001), a stark difference was found between the participants’ own vs. the anticipated professional groups’ power sensitivity (73.9% vs. 53.4%, *p* < 0.001). A hypothetical individual change for the worse through the power as social worker was rated significantly lower than all other items on the respective scale (61.5%, *p* < 0.001). Professionals rated the experience of stereotypical ideas and prejudices towards clients (78.5, 75.2%) to be significantly stronger than students (69.4, 67.4% all *p* < 0.001). For students, power sensitivity generally increased with semesters (*p* < 0.001), while it remained stable over professional years for social workers. Differences between fields of profession did not reach significance. In summary, both students and professionals emphasized the importance of power sensitivity, but seemed to show a self-serving bias if they compared themselves to their group – considering a possible corruption effect, this may at least be interpreted as problematic. We discuss room for improvement in terms of sensitization, whether in the context of further training (professionals) or curricula (students).

## 1 Introduction

As a human rights profession, social work aims to enable clients in difficult circumstances to lead a dignified life (1, 2) and to promote their participation in society in various ways (3–7). In this matter, a successful working relationship between social work professionals and their clients is essential. To fulfill their role, social workers have various means of power at their disposal. These include, among other things, residence determination in cases of child endangerment and decisions on granting or cancelling social, monetary, or educational support. The overarching goals are to benefit their clients and to carry out social mandates. Unfortunately, the asymmetrical nature of this professional relationship can be abused, with potentially negative consequences for those seeking support. A critical discourse on this topic is held regularly (8, 9), pointing out the unconscious nature of the matter.

Abuse of power in social work can occur at the individual, group or systemic level. Examples include institutional abuse of power, the misuse of sanctions or the malicious exploitation of knowledge advantages towards clients (10). The reasons for abuse of power are complex and multi-layered. On the one hand, the desire for power can be considered a basic human trait (11). Destructive forms of the use of power (i.e., abuse) lead to negative consequences for clients and may, in the long term, fall back on the person who abuses power. Empathy from those in positions of power can decrease, leading to a shift in focus to one’s own needs (egocentrism) and a sense of alienation from others (here: the clients). Ultimately, this can lead to immoral behavior (12) which is highly problematic in asymmetrical relationships.

On the other hand, a corrupting effect may facilitate the abuse of power. This very old assumption [“Power tends to corrupt, and absolute power corrupts absolutely” (13)] has been underpinned by social psychology experiments and leadership research since the 1950s. Paradoxically, a person’s belief that they are not susceptible to corruption can be considered a first step towards abusing power. Classic experiments such as Stanley Milgram’s obedience studies (14, 15) and the Stanford Prison Experiment (16) illustrate how easily people lose moral inhibitions under the influence of power (e.g., resulting in administration of supposed electric shocks, mistreatment of others). Muzafer Sherif’s vacation camp experiments (17) document how easily groups can become hostile when power and competition come into play. A modern replication of the Stanford Prison Experiment from 2006 also shows that these tendencies persist beyond historical contexts or the zeitgeist. The replication ultimately led to increasing acceptance of a tyrannical regime despite actively propagating humanitarianism (18).

Regardless of the cause, whether a basic human trait or a corruption effect, raising awareness of one’s own use of power and its consequences seems especially important in professions with asymmetrical relationships, such as social work. To date, two survey studies systematically assessed *power sensitivity* with the goal of developing standardized items that measure sensitivity in working practice (19, 20). Within these studies, power sensitivity was generally defined as a constitutive sensitivity towards the use of power (19). This comprises an awareness of one’s own status, potential mechanisms of corruption, the inherent potential of power, different perceptions of power use depending on one’s perspective, as well as the socio-psychological pitfalls of one’s own perception.

In both studies, a further distinction was made between two aspects of power sensitivity (19, 20): (a) *Basic* aspects included the relevance for working practice, how one’s own and the professional group’s power sensitivity was assessed (i.e., detection of possible self-esteem distortions), and whether/how relevant ethical attitudes in the professional context were intertwined with the use of power (21–23). (b) *Specific* aspects were derived directly from the general definition of power sensitivity – some of which have been shown to be specifically relevant for asymmetrical social interactions, particularly in psychology – and were then applied to the environment of social work (24–26). These specific aspects included sensitivity toward one’s status as a social worker, the risk of corruption and the potential for power to affect everyday working life. They also included divergent perceptions of power between social workers and clients and possible biases in perception that could influence interactions with clients, such as stereotypes and prejudices.

In the first study (19), social work professionals rated their power sensitivity in everyday working live at 7.9 out of 10 points. The same professionals rated the importance of adhering to ethical principles even higher, at 9.4 out of 10 points. Interestingly, participants rated their own power sensitivity higher than that of their professional group (*p* < 0.001). In light of the aforementioned corruption effect, this may at least be interpreted as problematic. As a result of the first study, a five-item numeric scale on specific power sensitivity was developed, along with an evidence-based action plan (19). The second study (20) surveyed both social work professionals and –students to further develop the scale within a larger sample. As a result, two partially overlapping scales for practical screening of specific power sensitivity were developed: one for social work professionals (nine items) and one for students (six items). In sum, students rated their specific power sensitivity as lower (69.6%) than professionals (73.1%).

So far, both previous studies focused on developing multi-item scales for specific power sensitivity along with single-item scales for basic power sensitivity. However, they only compared social work professionals and –students for some of these items due to a differing item-set between both groups. The present study aims to compare these groups on a larger scale. This cross-sectional survey uses common items both for 271 students and 414 professionals, covering basic and specific aspects of power sensitivity. It has not yet been examined whether there are general differences in basic and specific power sensitivity between both groups. The second aim of this study is to assess whether specific power sensitivity changes with increasing professional experience, such as length of studies, years of profession, and field of profession. Here, subgroup analyses open up the possibility to show trajectories of specific power sensitivity. In sum, we aim to answer the following research questions:

*Q1* – Does basic/specific power sensitivity differ between social work professionals and –students?

*Q2a* – Students: Does specific power sensitivity increase with rising semesters?

*Q2b* – Professionals: Does specific power sensitivity increase with increasing number of professional years? Does specific power sensitivity differ between fields of profession?

## 2 Materials and methods

### 2.1 Sample and study design

For this study, we conducted a Germany-wide, cross-sectional online questionnaire addressed to social work students and social work professionals. Questionnaires were completed from July 15 to September 15, 2024, via the SoSci platform (SoSci Survey GmbH). The survey captured two primary outcomes: (1) *basic* ratings on power sensitivity (e.g., general importance of the term; 4 items) and (2) *specific* ratings on power sensitivity (e.g., in a professional context; 5 items). Additional items included demographic and further variables. Please see section 2.2 for items and details.

Online questionnaires were disseminated via multiple routes: (1) institutions and/or their employees of social work: contact via e-mail (e.g., social services, child and adolescent psychiatric clinics, youth welfare facilities), (2) universities in Germany: contact via e-mail distribution lists for social work students, partly via student councils (e.g., University Duisburg-Essen, University of Applied Sciences and Arts (HAWK) Hildesheim/Holzminden/Göttingen, University of Applied Sciences and Arts Münster, University Eichstätt), (3) closed groups in social networks and platforms: postings (Facebook and LinkedIn), (4) professional associations: contact via e-mail (German Professional Association for Social Work, German Association for Social Work in Health Care).

In total, 829 persons entered data into the online survey. Data entered analysis if all items for *specific* ratings on power sensitivity were completed, which applied to a total of *N* = 685 out of 829 participants (82.6% completers). The survey was anonymous, and no data were collected that would allow conclusions to be drawn about an individual without considerable effort. Thus, obtaining an approval from the ethics committee was not necessary. All participants consented to the privacy policy at the beginning of the questionnaire.

### 2.2 Measurement

Besides the primary endpoints (see below), age and gender were recorded as demographic information. Specific information was collected depending on the participants’ occupational situation. Students were asked about their planned graduation (Bachelor’s vs. Master’s degree) and their semester. Social work professionals were asked about their years in the profession, and their area of professional activity (open response). Newly modified items and a shortened version of the questionnaire *Screening for Power Sensitivity* (20) were used to measure both primary endpoints.

1. *Basic* ratings on power sensitivity comprised four statements (items 1a to 1d; please see Table 1 for details). Participants provided ratings for these statements (e.g., 1a: “I think power sensitivity is important”) on a numeric scale from 0 to 100% with a grid spacing of 1%. Each numeric scale contained two opposed anchor points at 0% (“strongly disagree”) and at 100% (“strongly agree”). Items 1a to 1d based on items which had already been used in previous studies (19, 20). These items were not designed to be averaged into a common scale: they comprised two ratings on the participants’ own (perspective one) vs. their professional groups’ (perspective two) basic power sensitivity (see Table 1; items 1b and 1c).
2. *Specific* ratings on power sensitivity comprised five statements (items 2a to 2e; please see Table 1 for details), which were rated in the same way as described above (e.g., 2b: “The power I have as a social worker could change even me for the worse”). These items were part of the previously developed and validated scale on specific power sensitivity within the field of social work. They were designed to be averaged into a common scale, but initially contained partially differing items for social work students (Cronbach’s *α* = 0.74, original: 6 items) and –professionals [Cronbach’s α = 0.81, original: 9 items (20)]. Here, the scale was shortened and measured by the same five items for both groups to ensure comparability (see Table 1). Shortening the scale also led to a reduction in internal consistency (students: α = 0.70; professionals: α = 0.66). Please see the limitations section for further discussion.

**Table 1:** Translated questionnaire items (primary endpoints).

Single items: Basic ratings on power sensitivity	Answer options
1a: “I think power sensitivity is **important**.”	Numeric scale^1^
1b: “I consider my**self** power-sensitive.”	
1c: “I consider my professional **group** to be power-sensitive.”	
1d: “Professional **ethics** and my own principles are important to me in my work.”	

Five-item scale: Specific ratings on power sensitivity	Answer options
2a: “Power has an **effect**, even before it is applied.”	Numeric scale^1^
2b: “The power I have as a social worker could **change** even me for the worse.”	
2c: “The extent to which one’s own word is heard has a lot to do with one’s **position** in society or within a group.”	
2d: “I have already experienced that I had **stereotypical** ideas about the people I was addressing.”	
2e: “I have already experienced that I had **prejudices** about the people I was addressing.”	

All statements were rated on a ^1^numeric scale (percentage from 0 to 100% with a grid spacing of 1% containing two anchors: “strongly disagree” at 0%; “strongly agree” at 100%). Besides the primary endpoints, demographic information was recorded (please see section 2.2 for details).

### 2.3 Statistical analysis

We used SPSS® statistical software (version 30) for data analysis. Descriptive representation of the variables was accomplished using means (*M*), mean differences (*M_Diff_*), standard deviations (*SD*), frequencies (*Freq.*), and correlations (*r*^1^). For comparison of demographic and additional variables between both samples we used *t-*tests (e.g., age) along with corresponding effect sizes (Cohen’s d: *d_emp_*) and one Chi-squared test (gender).

For the first primary endpoint of this study (*basic* ratings on power sensitivity) analysis was performed on the single-item level (items 1a to 1d) via general linear model (GLM) for dependent measures. The participants’ ratings on the items were integrated as a four-stage within-subject factor. Occupational situation (“student” vs. “professional”) was integrated as two-stage between-subject factor. To map possible variations in pairwise differences between students and professionals for the single items, the interaction effect between both factors was tested together with the two main effects. Four additional pairwise comparisons (*t*-tests) were performed to validate a possible interaction effect between students and professionals for items 1a to 1d.

As second primary endpoint of this study, *specific* ratings on power sensitivity were compared between both samples for the combined scale comprising all five items with a single *t*-test (averaged items 2a to 2e; see section 2.2 for details). Differences between both groups were also analyzed on the single-item level for items 2a to 2e analogously to our first primary endpoint: We created a second GLM and integrated the participants’ ratings as five-stage within-subject factor. Again, the two main effects, interaction effect and five pairwise comparisons were tested for significance.

Additional within-group analyses were performed for both subgroups on the common scale of *specific* power sensitivity. (1) For social work students, we analyzed semester-wise differences on power sensitivity via UNIANOVA (eight subgroups: semesters 1 to ≥ 8) with pairwise comparisons between the semesters. (2) For social work professionals, the years in profession were categorized in five-year steps, leading to an overall of seven categories being analyzed analogously (UNIANOVA, seven subgroups: < 5 years to ≥ 30 years). Again, pairwise comparisons were used to analyze possible differences between these subgroups. Furthermore, an additional UNIANOVA was created to analyze differences between social work professionals depending on their professional field of activity (eight subgroups built on free responses; see results section for details on differentiation between the fields).

Due to *α*-error inflation, all pairwise comparisons reported here were adjusted within each model (GLMs/UNIANOVAs) using the Bonferroni method. A global adjustment over all models was not performed. Participants were allowed to skip individual items leading to a variation of included cases. Please see the degrees of freedom for each statistical test and further details in the results section.

## 3 Results

### 3.1 Sample and descriptive results

In sum, 414 social work professionals and 271 social work students participated in this study (total *N* = 685; please see Table 2 for an overview and correlations between descriptive variables). Of the total sample, *n* = 533 (77.8%) were female, *n* = 111 (16.2%) were male, *n* = 22 (3.2%) identified as diverse, and *n* = 19 (2.8%) gave no answer with regard to their gender. The subgroup of professionals had proportionally more male members (+6.1%), fewer female members (−3.4%) and fewer diverse members (−2.8%; χ^2^(df = 2, *N* = 666) = 7.35, *p* = 0.025). The overall mean age was *M* = 34.58 years (SD = 11.36). Students were *M_Diff_* = 11.93 years younger on average (*M* = 27.17, SD = 7.12) than professionals (*M* = 39.10, SD = 11.09; *t*(621) = 14.78, *p* < 0.001, *d_emp_* = 1.22). The overall scale of specific power sensitivity exceeded ratings of 75% for the total sample (*M* = 75.06; SD = 17.22). Differences on the global level were minimal and did not reach significance between professionals (*M* = 75.98, SD = 16.70) and students (*M* = 73.66, SD = 17.92, *M_Diff_* = 2.32, *t*(683) = 1.72, *p* = 0.085, *d_emp_* = 0.14).

**Table 2:** Correlations and descriptive results.

Variable	1	2	3	4	M ± SD/Freq.
Sociodemographic variables					
1. Professional vs. student	–				p: 414 s: 271
2. Gender (binary)	0.075	–			m: 111 f: 533
3. Age (years)	−0.510^***^	−0.172^***^	–		34.58 ± 11.36
4. Specific power sensitivity (%)	−0.066	−0.033	−0.048	–	75.06 ± 17.22

Correlations: * *p* < 0.05; ** *p* < 0.01; M = mean; SD = standard deviation; Freq. = frequency; professional = 1, student = 2; gender (male = 1, female = 2); specific power sensitivity = averaged ratings for the items 2a to 2e (see Table 1) from 0 to 100% (“strongly disagree” to “strongly agree”); *N* = 685 (gender: non-binary: n = 22, no answer: *n* = 19).

### 3.2 Basic power sensitivity in social work professionals vs. students

**Figure 1:**
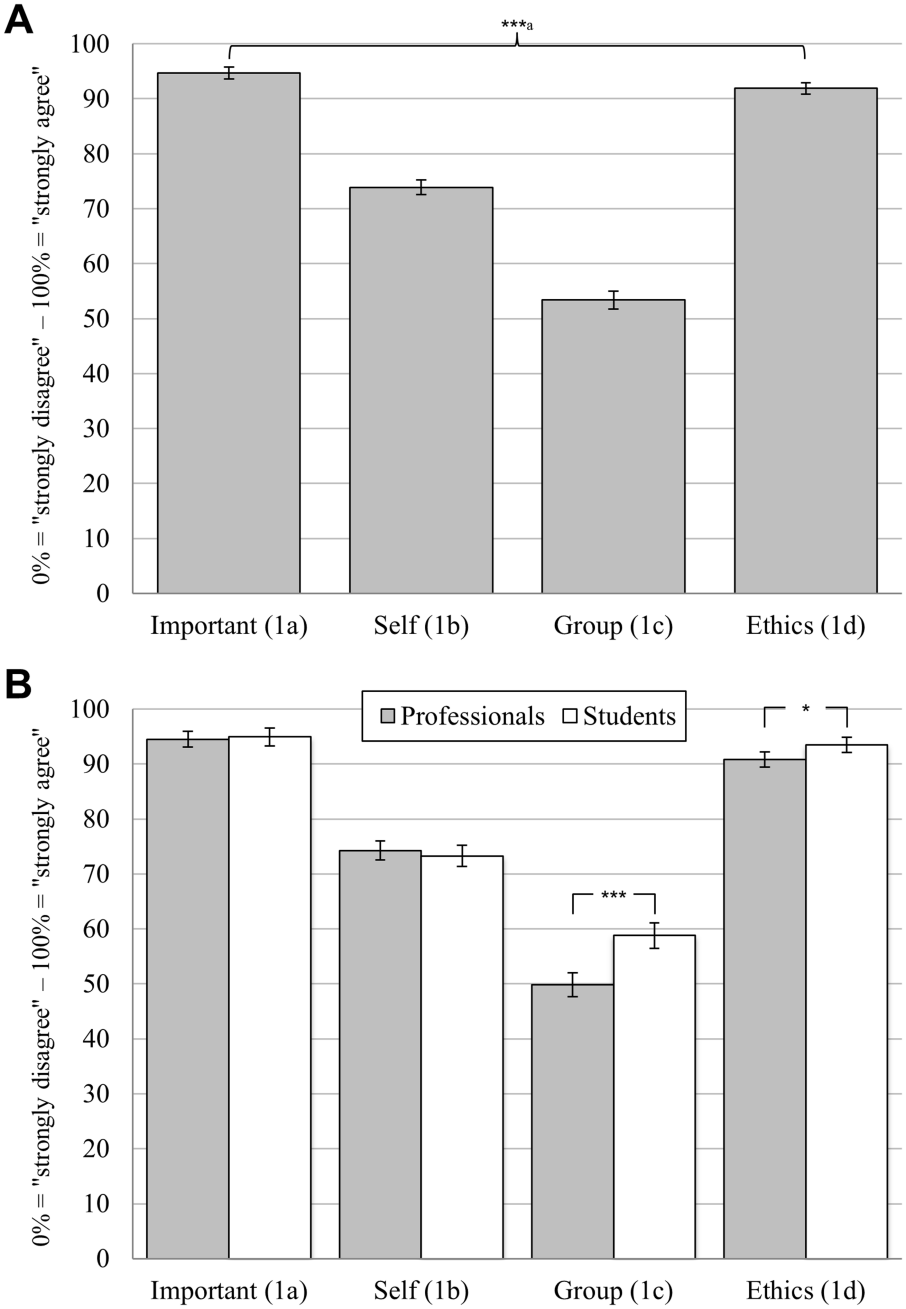
Ratings on basic power sensitivity on the single item level. Bonferroni corrected pairwise comparisons: *p < 0.05; ***p < 0.001; means with 95% confidence intervals; numeric scales from 0 to 100% with a grid spacing of 1% and two opposed anchor points at 0% (“strongly disagree”) and 100% (“strongly agree”; please see Table 1 for all item formulations). **(A)** Differences between items 1a to 1d for the whole sample (*N* = 672, *n* = 13 missing); ^a^all pairwise comparisons significant (*p* < 0.001). **(B)** Pairwise differences between social work professionals (*n* = 408) and -students (*n* = 264) for items 1a to 1d.

Out of *N* = 685 participants, 672 rated all four statements for basic power sensitivity (items 1a to 1d). Analysis revealed a significant dependent-measures effect for the total sample (GLM: *F*(3, 2010) = 1004.53, *p* < 0.001, partial η^2^ = 0.60). All six pairwise comparisons between items 1a to 1d reached significance (all *p* < 0.001; see Figure 1A for an overview, and Table 1 for all item formulations). The highest rating was found for (1) item 1a (“I think power sensitivity is important”; *M* = 94.69%, SD = 14.14), followed by (2) item 1d (“Professional ethics and my own principles are important to me in my work”; *M* = 91.88%, SD = 13.44), (3) item 1b (“I consider myself power-sensitive”; *M* = 73.89%, SD = 17.50) and (4) item 1c (“I consider my professional group to be power-sensitive”; *M* = 53.37%, SD = 21.62).

Both a significant between-subjects effect (GLM: *F*(1, 670) = 10.62, *p* = 0.001, partial η^2^ = 0.02) and a significant interaction effect was found (GLM: *F*(3, 2010) = 13.68, *p* < 0.001, partial η^2^ = 0.02), indicating general differences between professionals vs. students, as well as varying pairwise differences between both groups on the item-level. As shown in Figure 1B, corrected pairwise comparisons revealed two significant differences: (1) Students rated the importance of ethics and their own principles (item 1d; *M* = 93.51%, SD = 11.46) higher than professionals (*M* = 90.82, SD = 14.50, *t*(670) = 2.55, *p* = 0.044, *d_emp_* = 0.20). (2) Students rated their professional group to be more power-sensitive (item 1c; *M* = 58.79%, SD = 19.38) than professionals (*M* = 49.87%, SD = 22.29, *t*(670) = 5.33, *p* < 0.001, *d_emp_* = 0.42).

### 3.3 Specific power sensitivity in social work professionals vs. students

**Figure 2:**
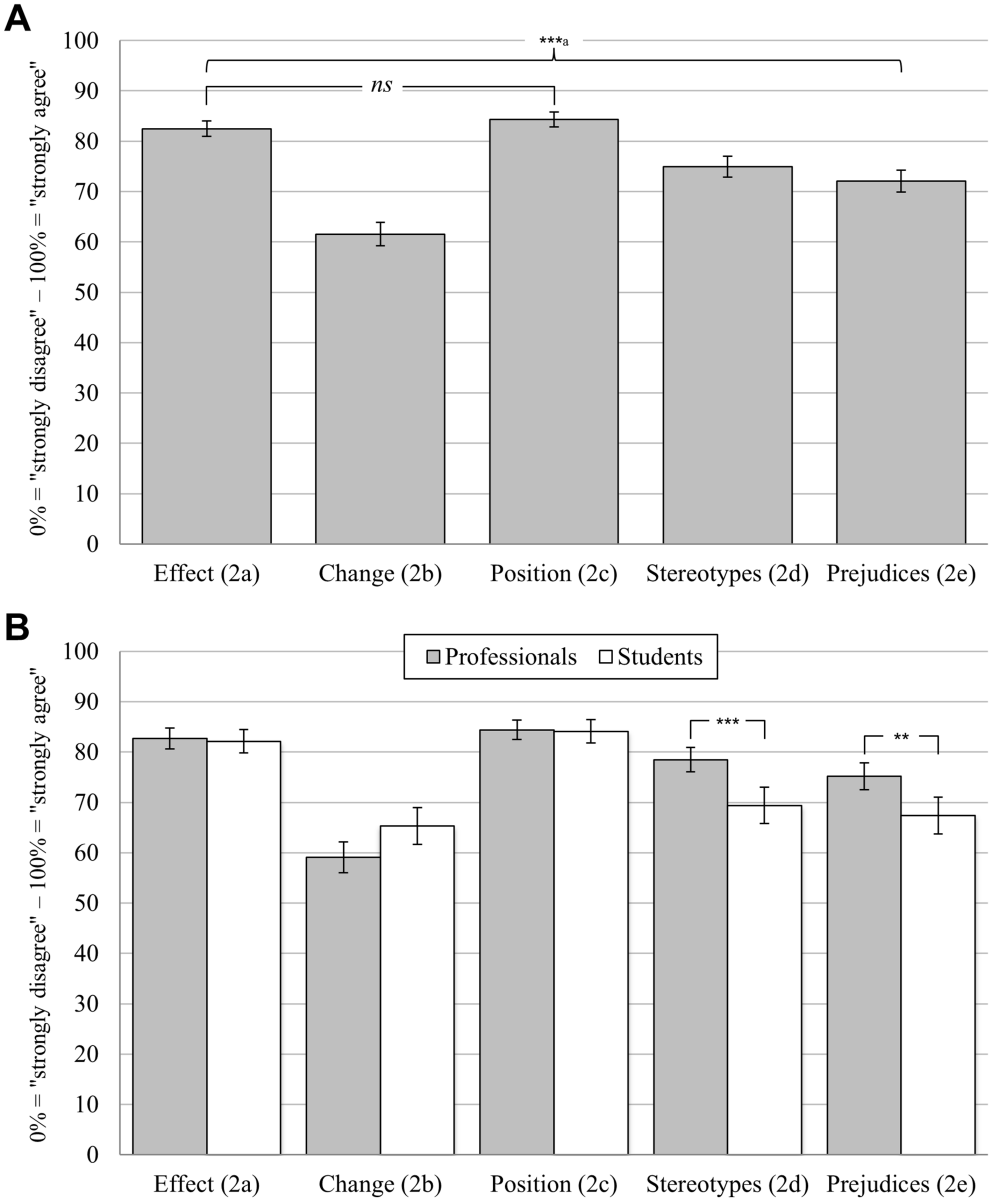
Ratings on specific power sensitivity on the single item level. Bonferroni corrected pairwise comparisons: ** p < 0.01; *** *p* < 0.001; means with 95% confidence intervals; numeric scales from 0 to 100% with a grid spacing of 1% and two opposed anchor points at 0% (“strongly disagree”) and 100% (“strongly agree”; please see Table 1 for all item formulations). **(A)** Differences between items 2a to 2e for the whole sample (*N* = 685); ^a^all pairwise comparisons significant (*p* < 0.001), except for the difference between 1a and 1c. **(B)** Pairwise differences between social work professionals (*n* = 414) and -students (*n* = 271) for items 2a to 2e.

For the single items 2a to 2e of the specific power sensitivity scale, a significant dependent-measures effect was found, indicating variation between the single items for the total sample (GLM: *F*(4, 2,732) = 110.28, *p* < 0.001, partial η^2^ = 0.14). Out of 10 pairwise comparisons, 9 reached significance (all *p* < 0.001; see Figure 2A for an overview, and Table 1 for all item formulations). In sum, item 2b (“The power I have as a social worker could change me for the worse”) was rated significantly lower than all other items (*M* = 61.54%, SD = 31.48, all *p* < 0.001). Both items 2a (“Power has an effect, even before it is applied”; *M* = 82.48%, SD = 20.67) and 2c (“The extent to which one’s own word is heard has a lot to do with one’s position in society or within a group”; *M* = 84.29%, SD = 19.52) were rated significantly higher than all the remaining items (all *p* < 0.001), but did not differ significantly from each other (*p* = 0.588).

Although no significant between-subjects effect was found (GLM: *F*(1, 683) = 2.97, *p* = 0.085, partial η^2^ < 0.01), a significant interaction effect indicated varying pairwise differences between professionals and students (GLM: *F*(4, 2,732) = 13.49, *p* < 0.001, partial η^2^ = 0.14). As illustrated in Figure 2B, corrected pairwise comparisons revealed significant differences between both groups in two cases: (1) Professionals stated to already have had stereotypical ideas about the people they were addressing (item 2d; *M* = 78.48%, SD = 25.41) to a higher degree than students did (*M* = 69.41%, SD = 30.04, *t*(683) = 4.25, *p* < 0.001, *d_emp_* = 0.33). (2) The same applied for prejudices about the people they were addressing: item 2e was also rated higher by professionals (*M* = 75.21%, SD = 27.61) than by students (*M* = 67.35%, SD = 30.83, *t*(683) = 3.48, *p* = 0.003, *d_emp_* = 0.27).

### 3.4 Within-group analysis: specific power sensitivity

**Figure 3:**
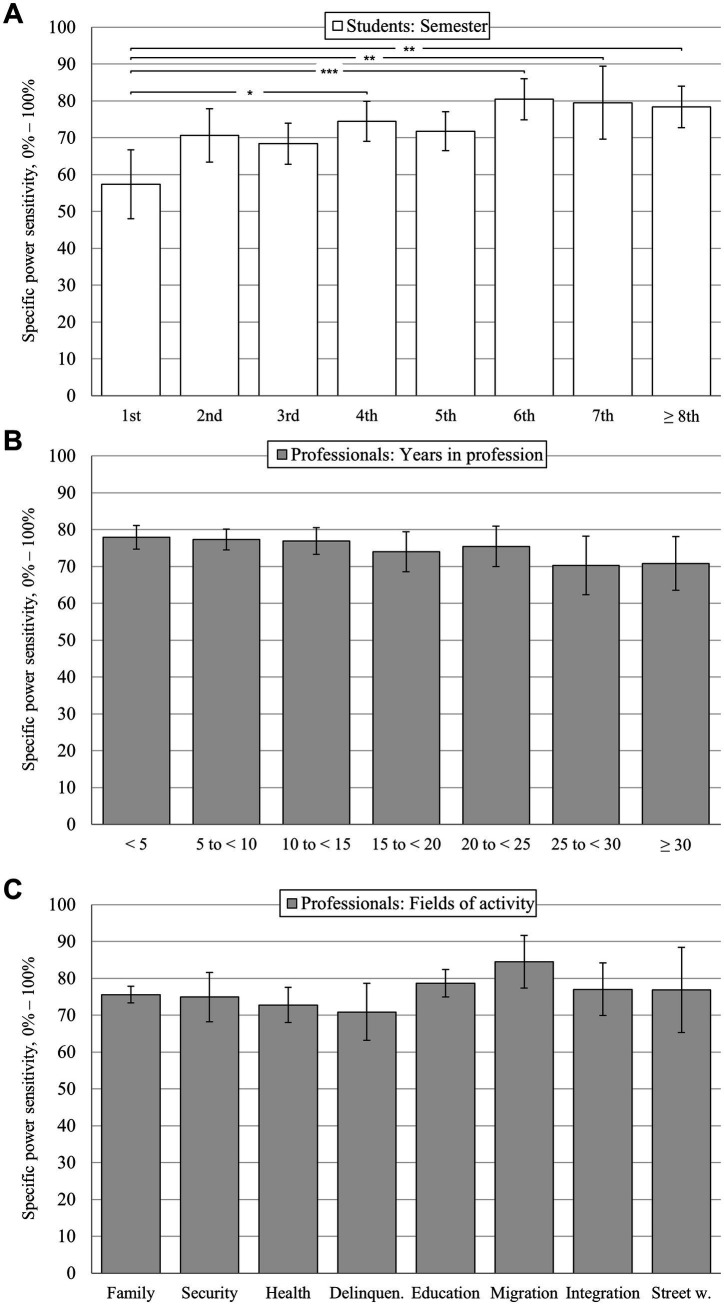
Ratings on specific power sensitivity on the global level. Bonferroni corrected pairwise comparisons: * p < 0.05; ** *p* < 0.05; *** *p* < 0.001; means with 95% confidence intervals; averaged numeric scale (items 1a to 1e) from 0 to 100% with a grid spacing of 1% and two opposed anchor points at 0% (“strongly disagree”) and 100% (“strongly agree”; please see Table 1 for all sub-item formulations). **(A)** Semester-wise differences for social work students (*N* = 236, subgroup range *n* = 41–13). **(B)** Differences between social work professionals assigned to subgroups depending on their years in profession (*N* = 409, subgroup range *n* = 115–18). **(C)** Differences between social work professionals assigned to subgroups depending on their field of activity (*N* = 395, subgroup range *n* = 163–15).

Out of *n* = 271 social work students, 238 were studying for a Bachelor’s degree of whom 236 provided information about their semester (Master’s degree: *n* = 32, missing: *n* = 1). For the within-group analysis, these *n* = 236 Bachelor students were selected and assigned to eight subgroups ranging from 1st semester to 8th and above. Subgroup size ranged from *n* = 13 (7th semester) to *n* = 41 (4th semester). Master’s students were excluded as semester-subgroups were too small (range from *n* = 1 to *n* = 10 across seven different semesters). Overall, power sensitivity was numerically lowest during the 1st Bachelor’s semester (*M* = 57.35%, SD = 19.07) and correlated positively with increasing semesters (*r* = 0.280, *p* < 0.001). The in-depth analysis revealed significant variation between the semesters, as illustrated in Figure 3A (UNIANOVA: *F*(7, 228) = 3.98, *p* < 0.001, partial η^2^ = 0.11). Corrected pairwise comparisons showed significant differences in 4 out of 28 pairs. Significance was reached for 1st vs. 4th semester (*M* = 74.47%, SD = 17.70, *p* = 0.031), 1st vs. 6th semester (*M* = 80.41%, SD = 17.49, *p* < 0.001), 1st vs. 7th semester (*M* = 79.49%, SD = 18.22, *p* = 0.025), and 1st vs. ≥ 8th semester (*M* = 78.37%, SD = 16.73, *p* = 0.003).

A total of *n* = 409 social work professionals provided information about their years in profession (range: 0.25–43 years; missing: *n* = 5). These were assigned to seven subgroups, each spanning 5 years (0 to < 5 years, 5 to < 10 years, etc.). Subgroup size ranged from *n* = 18 (25 to < 30 years) to *n* = 115 (5 to < 10 years). Power sensitivity was negatively correlated with increasing years in profession (*r* = −0.105, *p* = 0.033). The UNIANOVA did not reveal a significant variation between the subgroups (*F*(6, 402) = 1.36, *p* = 0.23, partial η^2^ = 0.02; all corrected pairwise comparisons ns, *p 0*.684 to 0.999; see Figure 3B). In sum, the minimal specific power sensitivity did not fall below an average of 70% for any subgroup.

Information about their professional field of activity was provided by *n* = 411 social work professionals (missing: *n* = 3). These free-text responses were then categorized using Pantuček-Eisenbacher’s well-established categorization system for professional fields in social work (27). To accomplish this, two experts assigned each free-text response to the appropriate pre-existing category. Any differences between the experts were discussed and resolved, if necessary. In sum, *n* = 13 responses could not be assigned clearly, and two fields comprised too small subgroups (“old people,” *n* = 2; “international−/development work,” *n* = 1). The remaining *n* = 395 responses were successfully assigned to eight subgroups (see Figure 3C), listed in descending order of size: (1) “children, young people, family” (*n* = 163), (2) “profession and education” (*n* = 71), (3) “health” (*n* = 63), (4) “basic security” (*n* = 31), (5) “delinquency” (*n* = 20), (6/7) “migration and integration”/“integration aid” (each *n* = 16), and (8) “community work and street work” (*n* = 15). Differences between these subgroups were minimal (UNIANOVA: *F*(7, 387) = 1.51, *p* = 0.161, partial η^2^ = 0.03; all corrected pairwise comparisons ns, *p 0*.342 to 0.999). The minimal specific power sensitivity was *M* = 70.88% (SD = 17.66, “delinquency”), compared to a maximum of *M* = 84.46% (SD = 14.61, “migration”).

## 4 Discussion

This cross-sectional questionnaire study aimed to gain insights into the perception and evaluation of power sensitivity. A total of 271 social work students and 414 -professionals were surveyed on this topic. First, we analyzed overall ratings and general differences between the groups for the single items of basic and specific power sensitivity. Second, we compared ratings for a five-item scale on specific power sensitivity within both subgroups (a) between Bachelor’s semesters (social work students) and (b) between years in profession as well as fields of profession (social work professionals).

### 4.1 Basic power sensitivity

The general importance of power sensitivity was rated high by the participants (94.7%) and did not differ between students and professionals. The importance of the participants’ own professional ethics/principles was rated similarly (91.9%). However, students rated this item significantly higher than professionals (+ 2.7%). Overall, these results suggest that the critical discourse on this matter (8, 9, 17) may also affect social workers in practice as well as during studies. Furthermore, the high level of agreement for both statements (> 90%) is in line with one previous study (19). On the one hand, this speaks for a currently successful and early sensitization.

On the other hand, a stark contrast was found concerning the ratings on the participants’ own (73.9%), vs. their group’s power sensitivity (53.4%). This finding is consistent with multiple studies in the context of social work (19, 20) and schools (23), and it may be interpreted as a self-serving bias. It could also be an empirical indication of the corruption effect and thus a precursor to the abuse of power, as observed within Stanley Milgram’s obedience studies (14, 15) and the Stanford Prison Experiment (16). One way to reduce a self-serving bias is to educate students and social workers that their own status and the associated power are not personal characteristics, but rather aspects of their professional role as social workers. This would ensure that the power available to them is geared toward benefiting their clients, thus expressing a professional relationship rather than being wrongly interpreted as a personal characteristic (10). Furthermore, it seems appropriate to educate (future) social workers on basic social psychological theories, such as the corruption effect, to increase power sensitivity. However, the difference between participants’ own vs. their group’s power sensitivity found here is significantly less pronounced among students, who rated the power sensitivity of their own group higher than professionals (though still lower than their own). One could speculate whether this is due to greater sensitization to this topic during studies or if the sensitivity just decreases over the years of work practice (please see 4.3).

### 4.2 Specific power sensitivity

Participants rated the likelihood that their power as social worker could change them for the worse significantly lower than all other items on the specific power sensitivity scale (61.5%). Again, this may allude to a typical bias for persons in positions of power (12, 26). They may perceive their own power as appropriate and manageable, while viewing the power of others more critically (see 4.1). In contrast, participants largely agreed with the statements that power even has an effect before it is applied (82.5%) and that one’s position in society/group is connected to enforcing one’s own word (84.3%). Both statements were rated significantly higher than all other items on the scale. These results suggest an awareness of the impact of power on everyday (working) life, both actively applied and as a passive status. Interestingly, both items regarding stereotypes and prejudices about their clients were rated > 72% by the total sample, but differed significantly between students and professionals. Social work professionals were more self-critical in acknowledging their own stereotypes and prejudices than students. The most obvious explanation would be the professionals’ work experience compared to students and the associated realization that prejudices and stereotypes occur and play a role in work practice. This can only be recognized if there is a certain amount of self-reflection regarding one’s own professional attitude (28). Nevertheless, students should be made more aware of stereotypical ideas and prejudices during their studies and reflect on them for their future work practice. This could possibly create an (even) greater awareness of such distortions in dealing with clients in the future.

### 4.3 Differences between semesters, professional years, and fields of profession

For Bachelor’s degree students, analysis revealed a positive correlation between specific power sensitivity and length of studies. During the 1st semester, it was rated lowest (57.4%), whereas significantly higher values were reached for 4th (74.5%) and from 6th to ≥ 8th semester (78.4–80.4%). Overall, students seemed to become increasingly sensitive to their power over clients. This could indicate that, in addition to developing specialist knowledge, students are also developing power-specific aspects of professional identity formation progressively during their studies. However, there still seems to be room for improvement in terms of a stronger professionalization of students regarding their power sensitivity. This could be incorporated into curricula: For example, since accreditation in 2016, two courses titled “Status and power relations” and “Power sensitivity in professional practice” have been successfully established at the HAWK Hildesheim/ Holzminden/ Göttingen within the study program “Bachelor of Social Work in Healthcare.” Both courses intend to promote power sensitivity by providing theoretical as well as evidence-based input. They contain practical exercises like role-playing. For example, a court trial may be reenacted, and the participants may reflect on their different positions of power (e.g., judge, defendant, prosecutor, defense attorney) afterward. Both courses are held on a regularly basis with 2 h per week per semester, the former during 3^rd^ semester as mandatory course, the latter during 6th semester as part of an elective subject. Such courses may facilitate the development of practical work skills (e.g., being able to assess own actions) and professional profiling (e.g., self-reflection skills, critical thinking).

For social work professionals, an increase in years of profession was negatively correlated with specific power sensitivity. The results showed a consistent, albeit minimal, reduction in values from 77.8 to 70.3%. Pairwise comparisons failed to reach significance, leading us to interpret power sensitivity as being largely stable or stagnant over the course of one’s career. However, there are indications that social work professionals with many years of professional experience are more likely to insist on their position of power and professional status than those who have not worked in the field as long (29). Professional burnout or institutional factors may also play a role here. One strategy to counteract a possible decline in power sensitivity is targeted, event-related training and further education that focuses on power sensitivity and the constructive use of power. Clearly, more empirical research is needed in this field. Encouragingly, differences in terms of specific power sensitivity across the fields of profession were minimal (all ratings > 70%). The lowest value was found for the coercive context with offenders (“delinquency”; 70.1%), the highest for working with migrants (84.5%). Overall, these results suggest that social workers seem to ascribe a high relevance to power sensitivity overall, regardless of their field of profession.

### 4.4 Limitations

First, power sensitivity was not assessed longitudinally in this cross-sectional questionnaire study. Therefore, differences between semesters (students) and professional years (social workers) were analyzed on an inter-subgroup level using a single survey. Consequently, differences between subgroups (e.g., individual semesters) may exist due to fundamental differences between them (e.g., personal traits), and thus may be misleading. It is, however, extremely improbable to find a largely consistent pattern such as increasing power sensitivity with each additional semester, as we found here. In sum, a longitudinal design would allow us to track intrapersonal changes over multiple semesters. This would come at the cost of feasibility and at the cost of a significantly smaller sample size due to potential dropouts over the years. Besides sample attrition, one problem could be the fear of a lack of anonymity in the event of repeated participation (e.g., through token-based assignment), which could reduce willingness to consistently participate in the study. It should also be noted at this point that the study design was susceptible to inclusion bias because participation was voluntary and most likely conducted by those who were interested in the topic. Second, most effect sizes found here are moderate (see results: GLMs). Due to our large overall sample size (*N* = 685) and also large subgroups, even small differences reached statistical significance despite Bonferroni correction. On the one hand, this can be criticized as overpowered study design. On the other hand, the large sample size enabled us to conduct further inter-subgroup analyses, which outweighs this disadvantage in our view. Third, the scale presented here for specific power sensitivity did not show a sufficient internal consistency for professionals (*α* = 0.66), but exclusively for students (α = 0.70). To this point, two established and reliable scales to assess power sensitivity do exist, but they have partially unique item-formulations for professionals (α = 0.81) and students [α = 0.74; (20)], which makes them less comparable. It would be worthwhile to further modify and empirically substantiate the scale presented here to achieve a better internal consistency with common items for both groups. The compromise was to select items that could be used in identical form for both students and professionals. The reduction in internal consistency observed in this study was partially caused by items 2d and 2e, which referred to stereotypical ideas and prejudices toward clients. While students tended to rate these items more cautiously, probably due to a lack of contact with clients, professionals gave more offensive ratings, probably due to their experience. In future versions, we plan to rewrite both statements in a way that is more universally applicable, as we did for items 2a to 2c. We expect this modified version to have a better internal consistency.

## 5 Conclusion

Power abuse in asymmetrical relationships as occurring in social work can have negative consequences both for clients and professionals. Therefore, power sensitivity is essential. As this study found, both social work students and social work professionals show good levels of sensitivity. However, the results also emphasize the need to raise awareness of power at various points, such as incorporating courses into curricula during studies and providing targeted, event-related training in work practice. On the one hand, differences between students and professionals highlight the importance of practical experience. On the other hand, these differences point to the need to link the theoretical content of training more closely with practical experience to raise awareness of power structures. One way to achieve this is by raising awareness of stereotypical thought patterns in order to reduce their impact in the context of social work interventions. Further research should focus on the mechanisms behind these differences and their impact on social work practice.

## Data Availability

The raw data supporting the conclusions of this article will be made available by the authors, without undue reservation.

## Footnotes

^1^Depending on scale level: Interval (Pearson), ordinal (Spearman) and binary (phi-coefficient).

## References

[1] IFSW, IASSW. Ethik in der Sozialen Arbeit - Darstellung der Prinzipien. (2004). Available online at: https://www.ethikdiskurs.de/fileadmin/user_upload/ethikdiskurs/Themen/Berufsethik/Soziale_Arbeit/Ethik_in_der_Sozialen_Arbeit.pdf (Accessed February 3, 2025)

[2] DBSH. Berufsethik des DBSH. Forum Soz [Internet]. (2014); Available online at: http://www.dbsh-hessen.de/uploads/tx_xpctypedownloadssimple/DBSH-Berufsethik-2015-02-08.pdf (Accessed February 3, 2025)

[3] Geyer C. Teilhabe 4.0.: Wie die Digitalisierung die Soziale Arbeit verändert. Soziale Arbeit. (2018) 67:457–64. doi: 10.5771/0490-1606-2018-12-457

[4] Polat A. Soziale Arbeit in der Migrationsgesellschaft - ihr (möglicher) Beitrag zu Teilhabe und Chancengerechtigkeit In: Gesemann F Filsinger D Münch S, editors. Handbuch Lokale Integrationspolitik. Wiesbaden: Springer Fachmedien (2024). 1–15.

[5] Rießen AV Henke S. Selbstbestimmte Teilhabe älterer Menschen durch ehrenamtliches Engagement: Chancen und Herausforderungen. Bl Wohlfahrtspfl. (2020) 167:173–6. doi: 10.5771/0340-8574-2020-5-173

[6] Spatscheck C. Inklusion und Soziale Arbeit: Teilhabe und Vielfalt Als Gesellschaftliche Gestaltungsfelder. Leverkusen-Opladen: Verlag Barbara Budrich (2017).

[7] Teubert A Rösner M. Teilhabe ermöglichen - Kompass für die Soziale Arbeit: Personenzentriert und wirkungsorientiert handeln. 1. Auflage ed. Stuttgart: Kohlhammer Verlag (2024).

[8] Sagebiel JB Pankofer S. Soziale Arbeit und Machttheorien: Reflexionen und Handlungsansätze. 2. aktualisierte Auflage ed. Freiburg: Lambertus-Verlag (2022).

[9] Staub-Bernasconi S. Umgang mit Machtquellen und Machtstrukturen als spezielle Handlungstheorien Sozialer Arbeit In: Soziale Arbeit als Handlungswissenschaft: Soziale Arbeit auf dem Weg zu kritischer Professionalität. 2nd ed. Stuttgart, Deutschland: utb GmbH (2018). 405–54.

[10] Misamer M. Machtsensibilität in der Sozialen Arbeit: Grundwissen Für Reflektiertes Handeln. 1st ed. Stuttgart: Kohlhammer Verlag (2023).

[11] Russell B Hermlin S. Formen der Macht. Köln: Anaconda Verlag (2009).

[12] Keltner D. The power paradox: How we gain and lose influence. Reprint ed. New York, USA: Penguin Publishing Group (2017).

[13] Acton JEED. Acton-Creighton correspondence | online library of liberty [internet]. (1887). Available from: https://oll.libertyfund.org/titles/acton-acton-creighton-correspondence (Accessed February 3, 2025)

[14] Milgram S. Obedience to authority: An experimental view. New York, USA: Harper & Row (1974).

[15] Milgram S. Behavioral study of obedience. J Abnorm Soc Psychol. (1963) 67:371–8. doi: 10.1037/h0040525, PMID: 14049516

[16] Haney C Banks C Zimbardo P. Interpersonal dynamics in a simulated prison. Int J Criminol Penol. (1973) 1:69–97.

[17] Sherif M. Experiments in group conflict. Sci Am. (1956) 195:54–8. doi: 10.1038/scientificamerican1156-54

[18] Reicher S Haslam SA. Rethinking the psychology of tyranny: the BBC prison study. Br J Soc Psychol. (2006) 45:1–40. doi: 10.1348/014466605X48998, PMID: 16573869

[19] Misamer M Hennecken L. Machtsensibilität in der Praxis Sozialer Arbeit - eine explorative Analyse. Evangelische Jugendhilfe. (2022) 99:194–201. doi: 10.17433/978-3-17-042186-8

[20] Misamer M. Machtsensibilität messen - ein Screening zur Selbsteinschätzung. Ein Blick auf den sensiblen Umgang mit Macht - mit Screening-Instrument für die Arbeitspraxis und das Studium. Forum sozial. (2024) 1:50–5.

[21] Witte EH. Theorien zur sozialen Macht. Hamburg: Universität Hamburg, Fak. für Erziehungswissenschaft, Psychologie und Bewegungswissenschaft, FB Psychologie, Arbeitsbereich Sozialpsychologie, vol. 30. Hamburg, Germany: Hamburger Forschungsberichte zur Sozialpsychologie (HaFoS) (2001).

[22] Wubbels T Brekelmans M. Two decades of research on teacher-student relationships in class. Int J Educ Res. (2005) 43:6–24. doi: 10.1016/j.ijer.2006.03.003, PMID: 40748484

[23] Misamer M. Macht und Machtmittel in der Schule: eine empirische Untersuchung. Lage: Jacobs Verlag (2019).

[24] Hogeveen J Inzlicht M Obhi SS. Power changes how the brain responds to others. J Exp Psychol Gen. (2014) 143:755–62. doi: 10.1037/a0033477, PMID: 23815455

[25] Keltner D Gruenfeld DH Anderson C. Power, approach, and inhibition. Psychol Rev. (2003) 110:265–84. doi: 10.1037/0033-295X.110.2.265, PMID: 12747524

[26] Scholl W. Einfluss nehmen und Einsicht gewinnen - gegen die Verführung der Macht. Wirtschaftspsychologie. (2007) 3:15–22.

[27] OBDS. Handlungsfelder der Sozialarbeit [Internet]. (2004) Available online at: http://www.pantucek.com/seminare/200609polizei/handlungsfelder.pdf (Accessed February 4, 2025)

[28] Bovha C Kontzi N Hahn J. Denkanstöße für die Soziale Arbeit. In: Anti-Bias-Netz In: Vorurteilsbewusste Veränderungen mit dem Anti-Bias-Ansatz. Freiburg im Breisgau: Lambertus (2016). 21–33.

[29] Misamer M Albrecht N. Zum Umgang mit Macht in der Arbeitspraxis: Ein machtsensibles Fachgespräch mit einer Führungskraft aus der Sozialen Arbeit. FORUM Sozial. (2023) 2:22–6.

